# Fungal air quality in hospital rooms: a case study in Tehran, Iran

**DOI:** 10.1186/2052-336X-11-30

**Published:** 2013-12-19

**Authors:** Faramarz Azimi, Kazem Naddafi, Ramin Nabizadeh, Mohammad Sadegh Hassanvand, Mahmood Alimohammadi, Shirin Afhami, Seyed Nejat Musavi

**Affiliations:** 1 Department of Environmental Health Engineering, School of Public Health, Tehran University of Medical Sciences, Tehran, Iran; 2 Center for Air Pollution Research (CAPR), Institute for Environmental Research (IER), Tehran University of Medical Sciences, Tehran, Iran; 3 Shariati Hospital, Tehran University of Medical Sciences, Tehran, Iran

**Keywords:** Fungal air quality, Hospital rooms, Indoor air monitoring

## Abstract

Fungi are usually presented in indoor environments and cause of many diseases. The aim of this descriptive study was to investigate the level of fungal contamination in hospital rooms. Sampling was conducted with an Andersen one-stage viable impactor (Quick Take-30) and counting plates containing a fungus-selective medium. A total of 120 air samples from ten hospital environments were performed. Airborne fungi concentrations were determined 72-120 hours after sampling. Total mean concentration of detected fungi in the hospital rooms was 55 ± 56 (mean ± SD) cfu/m^3^. The findings of the fungal concentration in the various hospital rooms revealed different levels of contamination: the lowest mean counts (37 ± 17 cfu/m^3^) were observed in NS 1 (Nursing Stations 1), and the highest (97 ± 217 cfu/m^3^) were reported in Orthopedics Operating Room (OOR). The most common fungal genus isolated were *Penicillium* (70%), *Aspergillus* (14%), *Cladosporium* (12%), *Alternaria* (2%) and others (2%). The obtained results showed that fungal concentrations in the present study were nearly high and these conditions should be considered as a risk factor for patients and other persons in the hospital.

## Introduction

In hospital facilities Indoor Air Quality (IAQ) is a critical factor in preventing infections. Unpleasant hospital IAQ may lead to hospital-acquired infections, sick hospital syndrome, and various occupational risks [1]. A large number of studies have showed that various percentages of hospital infections were caused by fungi, such as *Candida albicans* and diverse species of *Aspergillus*, *Cladosporium*, and *Penicillium*. [2-5]. Fungal air quality in the hospital environment affected by various factors, such as the presence of construction activity and a favorable microclimate. Since exposure to fungi can cause serious health problems, it is clearly essential, in the above-mentioned risk situations, to evaluate the level of contamination in the hospital environments and to use those evaluations to determine the risk of infection for patients and staff alike, in that the use of air conditioning systems does not provide complete protection against fungi [2,6-8]. Various studies have investigated the fungal air quality in hospital environments. Perdelli et al. [2] studied various environments in 10 hospitals and found the average concentration of airborne fungi was 19 ± 19 cfu/m^3^. Fungal air quality of three hospitals was studied in Greec by Panagopoulou et al., and reported that the median concentration of airborne fungi was ranged between 5.5 and 10.6 cfu/m^3^[6]. In other study conducted by Li and Hou [9], Bioaerosol characteristics were investigated in hospital clean rooms. They found that the concentrations of fungi varied from 0 to 319 cfu/m^3^.

The aim of the present study was to evaluate the level of airborne fungal contamination in various hospital rooms in Shariati Hospital, Tehran, Iran.

## Materials and methods

### Hospital environments

The present study was conducted in ten rooms of Shariati Hospital in city of Tehran, Iran. The air sampling occurred during January to April 2012, and 120 samples were examined. In the hospital, ten rooms in two floors (first and second) were studied and categorized into: Operating Rooms (OR) (8 rooms: General Operating Room 1(GOR1), Orthopedics Operating Room (OOR), Nerves Operating Room (NOR), General Operating Room 2 (GOR2), Urology Operating Room (UOR), Women Operating Room (WOR), Emergency Operating Room (EOR), Maxillofacial Operating Room (MOR)) and Nursing Stations (NS) (2 stations).

### Air sampling

Sample collection was performed in respiratory height (about 1.5 m) for 2 minutes using an Andersen one-stage viable impactor (Quick Take-30, SKC, USA) at an airflow rate of 28.3 L/min [10-12], and SKC BioStage single-stage viable cascade impactors equipped with 100 mm diameter Petri dishes containing Sabouraud dextrose agar medium, supplemented with chloramphenicol. Before the air sampling, the head was cleaned with 70% alcohol [13]. The Petri dishes were closed and delivered to the Central Laboratory of Public Health School, Tehran University of Medical Sciences. Indoor air temperature and relative humidity were measured using a digital PHB-318.

### Fungi incubation and identification

This step was according to other study [2] in brief, the plates were incubated at 25°C and counted after 72-120 hours and reported as colony forming unit (cfu/m^3^). When suspect colonies were detected, they were isolated with plates containing Sabouraud plus chloramphenicol medium. The incubation temperature was 25°C. The airborne fungi concentrations were identified using both microscopic and macroscopic methods for each colony isolated.

### Data analysis

Descriptive statistics by the SPSS software version 20 are used for data analysis in this study.

## Results

Relative humidity and temperature of indoor air ranged from 16% to 42% and 19°C to 26°C, respectively. Total mean of airborne fungal concentration was 55 ± 56 cfu/m^3^. Analysis of the fungal concentration in the various types of rooms shown different levels of contamination: the lowest mean values (37 ± 17 cfu/m^3^) were detected in Nursing Stations 1 (NS 1), and the highest (97 ± 217 cfu/m^3^) were detected in Orthopedics Operating Room (OOR) (Table 1).

**Table 1:** Descriptive statistics for total detected airborne fungi and environment conditions of hospital rooms

**Floor**	**Hospital rooms**	**No. of Samples**	**Mean (cfu/m**^**3**^**) (Min-Max)**	**SD**	**Indoor environment conditions (Mean)**	
					**T (°C)**	**RH (%)**
First	GOR1^1^	12	43(18-159)	52	22	31
	OOR^2^		97(18-707)	217	22	30
	NOR^3^		40(18-71)	18	23	27
	GOR2^4^		42(18-88)	25	23	27
	UOR^5^		58(18-300)	83	22	29
	NS1^6^		37(18-71)	17	23	26
Second	WOR^7^		53(18-283)	43	23	25
	EOR^8^		71(18-141)	43	23	26
	MOR^9^		47(18-88)	26	24	28
	NS2^10^		62(18-141)	37	26	26
Total		120	55(18-707)	56	23(19-26)	27(16-42)

^1^GOR: General Operating Room.

^2^OOR: Orthopedics Operating Room.

^3^NOR: Nerves Operating Room.

^4^GOR2: General Operating Room 2.

^5^UOR: Urology Operating Room.

^6^NS 1: Nursing Stations1.

^7^WOR: Women Operating Room.

^8^EOR: Emergency Operating Room.

^9^MOR: Maxillofacial Operating Room.

^10^NS 2: Nursing Stations 2.

Table 2 presents the concentration of airborne fungi genera in various hospital rooms. The results showed the dominant fungal species were *Penicillium* (70%), *Aspergillus* (14%), *Cladosporium* (12%), *Alternaria* (2%) and others (2%). Also the highest mean concentration of *Penicillium*, *Aspergillus*, *Cladosporium* and *Alternaria* were detected in OOR, NS 1, NS 1 and OOR, respectively.

**Table 2:** **Concentration of airborne fungi genera in various hospital rooms (Mean ± SD) in cfu/m**^**3**^

**Floor**	**Hospital rooms**	**Fungi genera**				
		***Penicillium***	***Aspergillus***	***Cladosporium***	***Alternaria***	**Other**
First	GOR1^1^	44 ± 57	18 ± 1	-	-	-
	OOR^2^	144 ± 276	35 ± 1	18 ± 1	35 ± 1	-
	NOR^3^	33 ± 21	35 ± 1	35 ± 1	-	-
	GOR2^4^	46 ± 32	90 ± 1	-	-	35 ± 1
	UOR^5^	85 ± 108	36 ± 1	-	-	-
	NS1^6^	38 ± 21	106 ± 1	18 ± 1	-	-
Second	WOR^7^	65 ± 107	18 ± 1	15 ± 1	-	53 ± 1
	EOR^8^	51 ± 41	28 ± 9	35 ± 12	-	-
	MOR^9^	39 ± 19	53 ± 1	53 ± 49	-	18 ± 1
	NS2^10^	41 ± 35	53 ± 1	62 ± 62	18 ± 1	-
Total mean (first and second floor)		59 ± 34	47 ± 30	34 ± 18	27 ± 12	35 ± 18

“-” means not detected.

^1^GOR: General Operating Room.

^2^OOR: Orthopedics Operating Room.

^3^NOR: Nerves Operating Room.

^4^GOR2: General Operating Room 2.

^5^UOR: Urology Operating Room.

^6^NS 1: Nursing Stations1.

^7^WOR: Women Operating Room.

^8^EOR: Emergency Operating Room.

^9^MOR: Maxillofacial Operating Room.

^10^NS 2: Nursing Stations 2.

**Figure 1:**
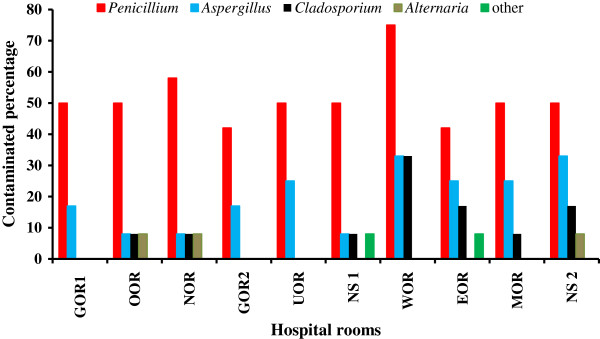
Percentage of hospital environments contaminated by various genera of fungi.

The degree of contamination by the fungal genera identified was calculated in percentage terms for each type of hospital room (Figure 1). Overall, the genus least frequently encountered was *Alternaria,* which displayed its maximum value of 8%. *Penicillium* species were detected in 75% of the WOR monitored, but contamination by these species was less frequent in EOR and GOR2 and these species had a more uniform distribution in the various locations; it was documented in approximately one-half of the tested areas in each type of environment. *Aspergillus* organisms were detected in 33% of WOR and NS2 and 25% of EOR, MOR and UOR rooms. Also, *Cladosporium* species were detected in 33% of WOR rooms.

## Discussion

To investigate the fungal air quality, 120 samples were collected from ten rooms of Shariati hospital. The results showed that total concentration of detected fungi in the hospital rooms was 55 ± 56 (mean ± SD) cfu/m^3^. Also the obtained results showed the most frequent fungal genus isolated were *Penicillium*, *Aspergillus* and *Cladosporium* that is consistent with other studies [2,14,15].

Fungal air quality in hospital environments was investigated by the numerous researchers. In a study carried out by Sautour et al., in France indoor fungal contamination was conducted during 18-month. They found that the average concentration of viable fungi in indoor air was 4.2 cfu/m^3^ and the most frequently detected airborne fungi were *Penicillium* spp. (27- 38%) and *Aspergillus* spp [16]. Thus the average concentration of fungi reported in their study is much lower than those detected in the present study. Fungal contamination (*Penicillium* and *Aspergillus*) of hospital rooms has been investigated in Lithuania and air borne fungi were ranged from 26 to 78 cfu/m^3^[14].

The results of the present study show various levels of contamination in all the hospital rooms, even though all areas are equipped with air conditioning systems. Such contamination may be caused or exacerbated by a range of factors, such as noncompliance with procedural norms (e.g., the frequent opening of doors among the operating theater and the outer environment) and inefficient operation or inadequate maintenance of the air conditioning system, which can allows unfiltered outside air to enter the operating theater. Overall, the mean concentrations of airborne fungi measured in the present study were higher than those recorded in other studies [6-8]. Moreover, our results show that the concentration of airborne fungi is significantly high in rooms and seems this concentration is important risks factor for patients and staff in these rooms. According our observations opening doors and windows are the most important factors that cause these high concentrations of airborne fungi in the rooms. Construction and demolition at the hospital campus during the study is other factor to high concentration of airborne fungi.

## Conclusion

In hospital environments indoor air quality is an important factor in preventing infections. Levels of airborne fungal contamination in various hospital rooms in Shariati Hospital, Tehran, Iran have been investigated. The results showed that fungal concentrations were high and these conditions should be considered as a risk factor for patients and other persons in the hospital.

## Competing interests

The authors declare that they have no competing interests.

## Authors’ contributions

All authors have made extensive contribution throughout the completion of this manuscript. All authors read and approved the final manuscript.

## References

[B1] Wan GH Chung FF Tang CS Long-term surveillance of air quality in medical center operating rooms Am J Infect Control 2011 11 302 308 10.1016/j.ajic.2010.07.006 21256628

[B2] Perdelli F Cristina ML Spagnolo AM Dallera BS Ottria G Grimaldi M Fungal contamination in hospital environments Infect Control Hosp Ep 2006 11 44 47 10.1086/499149 16418986

[B3] Faure O Fricker-Hidalgo H Lebeau B Ambroise-Thomas P Eight-year surveillance of environmental fungal contamination in hospital operating rooms and haematological units J Hosp Infect 2002 11 155 160 10.1053/jhin.2001.1148 11846544

[B4] Fox BC Chamberlin L Kulich P Rae EJ Webster LR Heavy contamination of operating room air by Penicillium species. Identification of the source and attempts at decontamination Am J Infect Control 1990 11 300 306 10.1016/0196-6553(90)90229-L 2135636

[B5] Panagopoulou P Filioti J Farmaki E Avgi MM Roilides E Filamentous fungi in a tertiary care hospital: environmental surveillance and susceptibility to antifungal drugs Infect Control Hosp Ep 2007 11 60 67 10.1086/508832 17230389

[B6] Panagopoulou P Filioti J Petrikkos G Giakouppi P Anatoliotaki M Farmaki E Environmental surveillance of filamentous fungi in three tertiary care hospitals in Greece J Hosp Infect 2002 11 185 191 10.1053/jhin.2002.1298 12419271

[B7] Rainer J Peintner U Poder R Biodiversity and concentration of airborne fungi in a hospital environment Mycopathologia 2001 11 87 97 10.1023/A:1007273131130 11265167

[B8] Molina RT Garijo MA Munoz RA Palacios IS Pollen and spores in the air of a hospital out-patient ward Allergol Immunopath 2002 11 232 238 10.1016/S0301-0546(02)79126-X 12199968

[B9] Li CS Hou PA Bioaerosol characteristics in hospital clean rooms Sci Total Environ 2003 11 169 176 10.1016/S0048-9697(02)00500-4 12670766

[B10] Naddafi K Jabbari H Hoseini M Nabizadeh R Rahbar M Younesian M Investigation of indoor and outdoor air bactrial density in Tehran subway system Iranian J Environ Health Sci Eng 2011 11 383 388

[B11] Obbard JP Fang LS Airborne concentrations of bacteria in a hospital environment in Singapore Water Air Soil Pollut 2003 11 333 341 10.1023/A:1022973402453

[B12] Yang CS Heinsohn PA Sampling and analysis of indoor microorganisms 2007 Hoboken, New Jersey: John Wiley and Sons, Inc

[B13] Hoseini M Jabbari H Naddafi K Nabizadeh R Rahbar M Yunesian M Jaafari J Concentration and distribution characteristics of airborne fungi in indoor and outdoor air of Tehran subway stations Aerobiologia 2012 1 9

[B14] Gorny RL Dutkiewicz J Bacterial and fungal aerosols in indoor environment in Central and Eastern European countries Ann Agric Environ Med 2002 11 17 23 12088392

[B15] Tormo-Molina R Gonzalo-Garijo MA Fernandez-Rodriguez S Silva-Palacios I Monitoring the occurrence of indoor fungi in a hospital Rev Iberoam Micol 2012 11 227 234 10.1016/j.riam.2012.04.002 22554822

[B16] Sautour M Sixt N Dalle F LOllivier C Calinon C Fourquenet V Prospective survey of indoor fungal contamination in hospital during a period of building construction J Hosp Infect 2007 11 367 373 10.1016/j.jhin.2007.09.013 18037534

